# National Public Health Week — April 3–9, 2017

**DOI:** 10.15585/mmwr.mm6612a8

**Published:** 2017-03-31

CDC joins the American Public Health Association (APHA) in celebration of National Public Health Week, April 3–9, 2017. Since 1995, APHA has led the observance of National Public Health Week during the first full week of April. The week recognizes the impact of public health on the health of the nation. The 2017 observance focuses on making the United States the Healthiest Nation in One Generation by 2030 by spotlighting the importance of prevention, employing successful strategies for collaboration, and promoting the critical role of a strong public health system. 

In conjunction with this year’s observance, CDC is partnering to promote APHA’s National Public Health Week themes, events, tools, and resources. Additional information is available at http://www.nphw.org/.

